# The association between *CDH1* promoter methylation and patients with ovarian cancer: a systematic meta-analysis

**DOI:** 10.1186/s13048-016-0231-1

**Published:** 2016-04-11

**Authors:** Qiang Wang, Bing Wang, Yun-mei Zhang, Wei Wang

**Affiliations:** Obstetrics and Gynecology Department, The Second Hospital of Jilin University, Changchun, 130041 China; Plastic Surgery Department, China-Japan Friendship Hospital Affiliated Jilin University, Changchun, 130033 China; Obstetrics and Gynecology Department, The Second People’s Hospital of Dunhua, Dunhua, 133700 China; Radiology Department, The First Hospital of Jilin University, Changchun, 130041 China

**Keywords:** CDH1 promoter, Methylation, Ovarian cancer, LMP

## Abstract

**Background:**

The down-regulation of E-cadherin gene (*CDH1*) expression has been regarded as an important event in cancer invasion and metastasis. However, the association between *CDH1* promoter methylation and ovarian cancer remains unclear. A meta-analysis was conducted to evaluate the potential role of *CDH1* promoter methylation in ovarian cancer.

**Methods:**

Relevant articles were identified by searches of PubMed, EMBASE, Cochrane Library, CNKI and Wanfang databases. The pooled odds ratio (OR) and corresponding 95 % confidence interval (CI) were calculated to assess the strength of association.

**Results:**

Nine studies were performed using the fixed-effects model in this study, including 485 cancer tissues and 255 nonmalignant tissues. The findings showed that *CDH1* promoter methylation had an increased risk of ovarian cancer in cancer tissues (OR = 8.71, *P* < 0.001) in comparison with nonmalignant tissues. Subgroup analysis of the ethnicity showed that the OR value of *CDH1* methylation in Asian population subgroup (OR = 13.20, *P* < 0.001) was higher than that in Caucasian population subgroup (OR = 3.84, *P* = 0.005). No significant association was found between ovarian cancer and low malignant potential (LMP) tumor (*P* = 0.096) among 2 studies, and between *CDH1* promoter methylation and tumor stage and tumor histology (all *P* > 0.05). There was not any evidence of publication bias by Egger’s test (all *P* > 0.05).

**Conclusions:**

*CDH1* promoter methylation can be a potential biomarker in ovarian cancer risk prediction, especially Asians can be more susceptible to *CDH1* methylation. However, more studies are still done in the future.

## Background

Ovarian cancer, the most lethal tumor in gynecologic cancers, is the fifth most cause of cancer-related deaths among women. According to cancer statistics, approximately 21,290 women will be diagnosed and 14,180 will die due to ovarian cancer in the United States in 2015 [1]. Among ovarian cancer, serous ovarian carcinoma is the most common histotype and only less than 20 % of ovarian cancer can be detected early due to the lack of effective early detection and accurate diagnosis methods [2]. More than 80 % of ovarian cancer patients at advanced stages relapse [3]. While the overall 5-year survival rate is only 31 % [4].

Epigenetic alterations (DNA methylation, histone modifications, nucleosome positioning and non-coding RNAs) are identified to be strongly associated with cancer [5]. DNA methylation is an important mechanism of epigenetic variability involved in gene expression, which plays key roles in the development of cancer [6–8]. Aberrant methylation of CpG islands of the promoter regions is the major alternative to accomplish tumor suppressor gene (TSG) silencing [9–11]. *CDH1*, a tumor suppressor gene, also called as epithelial cadherin (E-cadherin) and cadherin-1, is located on 16q23 [12]. *CDH1*, a member of the cadherin family, plays an important role in epithelial cell-cell adhesion and in maintaining normal tissue architecture [13]. The reduction of *CDH1* expression may involve in invasion and metastasis of several cancers [13–15].

However, the association between *CDH1* promoter methylation and ovarian cancer remains to be certified. In this study, we performed a meta-analysis to evaluate the relationships between ovarian cancer tissues and nonmalignant ovarian tissues and Low malignant potential (LMP) tumor tissues. In addition, we also assess the relationship between *CDH1* promoter methylation and clinicopathological features in ovarian cancer.

## Methods

### Literature search and selection criteria

A systematic literature search was performed in PubMed, EMBASE, Cochrane Library, CNKI and Wanfang databases, using the following keywords and search items: (CDH1 OR E-cadherin OR cadherin 1) AND (ovarian OR ovary) AND (cancer OR carcinoma OR tumor) AND methylation. The search updated until December 25. 2015. Moreover, a manual search of the references was also conducted to identify the potentially additional articles.

For eligible studies, studies must meet the following criteria: (1) all patients were diagnosed for primary ovarian cancer; (2) the study was about *CDH1* promoter methylation and ovarian cancer; (3) study must have sufficient data about the frequencies of *CDH1* promoter methylation to assess to the relationship between *CDH1* promoter methylation and ovarian cancer; (4) only the most recent paper or the most complete one was selected to avoid duplicated publications. Study was excluded if it did not meet the inclusion criteria above.

### Data extraction

For each eligible study, the following information were extracted: the first author’s name, publication year, methylation region, country, ethnicity, the method of methylation detection, type of control, the number of methylation, the sample size, clinicopathological parameters, such as the number of tumor stage, the number of tumor histology, etc. Nonmalignant ovarian tissues were defined as controls, including benign disease, normal tissues or adjacent normal tissues. Low malignant potential (LMP) tumors were also served as a single control group.

### Statistical analysis

Meta-analysis was conducted using the STATA software (version 12.0, Stata Corporation, College Station, TX, USA). The pooled odds ratios (OR) and 95 % confidence interval (95 % CI) were calculated to evaluate the association between *CDH1* promoter methylation and ovarian cancer risk. Between-study heterogeneity was examined using the Cochran’s Q test and I^2^ statistic [16]. If I^2^ < 50 % and *p* ≥ 0.1 were considered as a measure of lack heterogeneity, a fixed-effects model was applied; otherwise, the random-effects model was used [17, 18]. Publication bias was assessed by using Egger’s linear regression test [19].

## Results

### Study characteristics

**Fig. 1:**
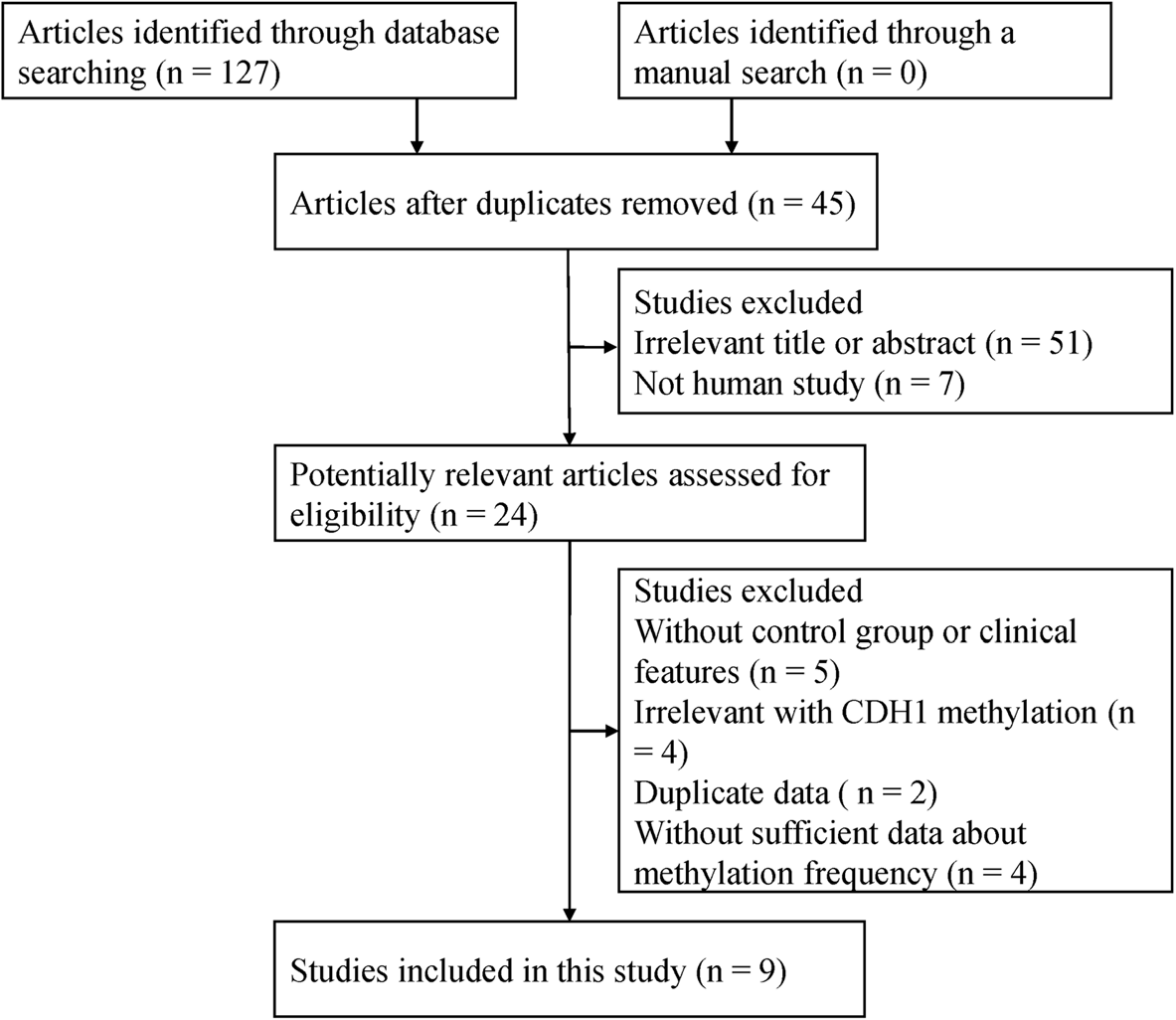
Flow diagram of the literature search strategy

One hundred twenty-seven potentially relevant articles were initially identified by the databases above. These studies were further selected based on the inclusion criteria. Finally, a total of 9 studies met the inclusion criteria were included in the current meta-analysis (Fig. 1). The methylation region of these studies was promoter. Among these studies, 8 studies used methylation-specific polymerase chain reaction (MSP) and 1 study used methylation specific headloop suppression PCR (MSHSP). There were two control groups, including nonmalignant control with 8 studies and LMP control with 2 studies. 8 studies evaluated the association between *CDH1* promoter methylation and ovarian cancer risk, 4 studies evaluated the relationship between *CDH1* and tumor histology, and 3 studies assessed the relationship between *CDH1* and tumor stage. The main characteristics of included studies were listed in Table 1 [20–26].

**Table 1:** The main characteristics of included studies in this meta-analysis

First author	Region	Country	Race	Method	Sample	Control	Case	Control	Stage 1-2	Stage 3-4	Serous	Non-serous
							M/N	M/N	M/N	M/N	M/N	M/N
Rathi 2002 [35]	Promoter	USA	Caucasians	MSP	Tissue	NMT	14/49	2/39	-	-	-	-
Makarla 2005 [21]	Promoter	USA	Caucasians	MSP	Tissue	NMT	6/23	4/39	-	-	2/9	3/13
Makarla 2005 [21]	Promoter	USA	Caucasians	MSP	Tissue	LMP	6/23	4/23	-	-	-	-
Yuecheng 2006 [26]	Promoter	China	Asians	MSP	Tissue	NMT	34/80	0/34	-	-	-	-
Shen 2007 [23]	Promoter	China	Asians	MSP	Tissue	NMT	18/63	1/30	2/22	16/41	9/34	9/29
Montavon 2012 [34]	Promoter	Australia	Caucasians	MSHSP	Tissue	NMT	17/78	1/5	-	-	-	-
Bhagat 2013 [20]	Promoter	India	Asians	MSP	Tissue	NM	31/86	2/34	8/23	23/63	17/44	7/25
Bhagat 2013 [20]	Promoter	India	Asians	MSP	Tissue	LMP	31/86	2/14	-	-	-	-
Wu 2014 [25]	Promoter	China	Asians	MSP	Tissue	NMT	32/50	-	7/12	25/38	25/35	7/15
Moselhy 2015 [22]	Promoter	Saudi Arabia	Asians	MSP	Tissue	NMT	12/18	8/32	-	-	-	-
Sun and Zhang 2015 [24]	Promoter	China	Asians	MSP	Tissue	NMT	15/38	1/42	-	-	-	-

*MSP* Methylation Specific PCR, *MSHSP* Methylation specific headloop suppression PCR, *NMT* nonmalignant tissues, *LMP* low malignant potential tumor, “-” indicates data not available, *M* stands for the number of methylation positive, *N* stands for the number of the total samples

### The association between *CDH1* promoter methylation and OC risk

**Fig. 2:**
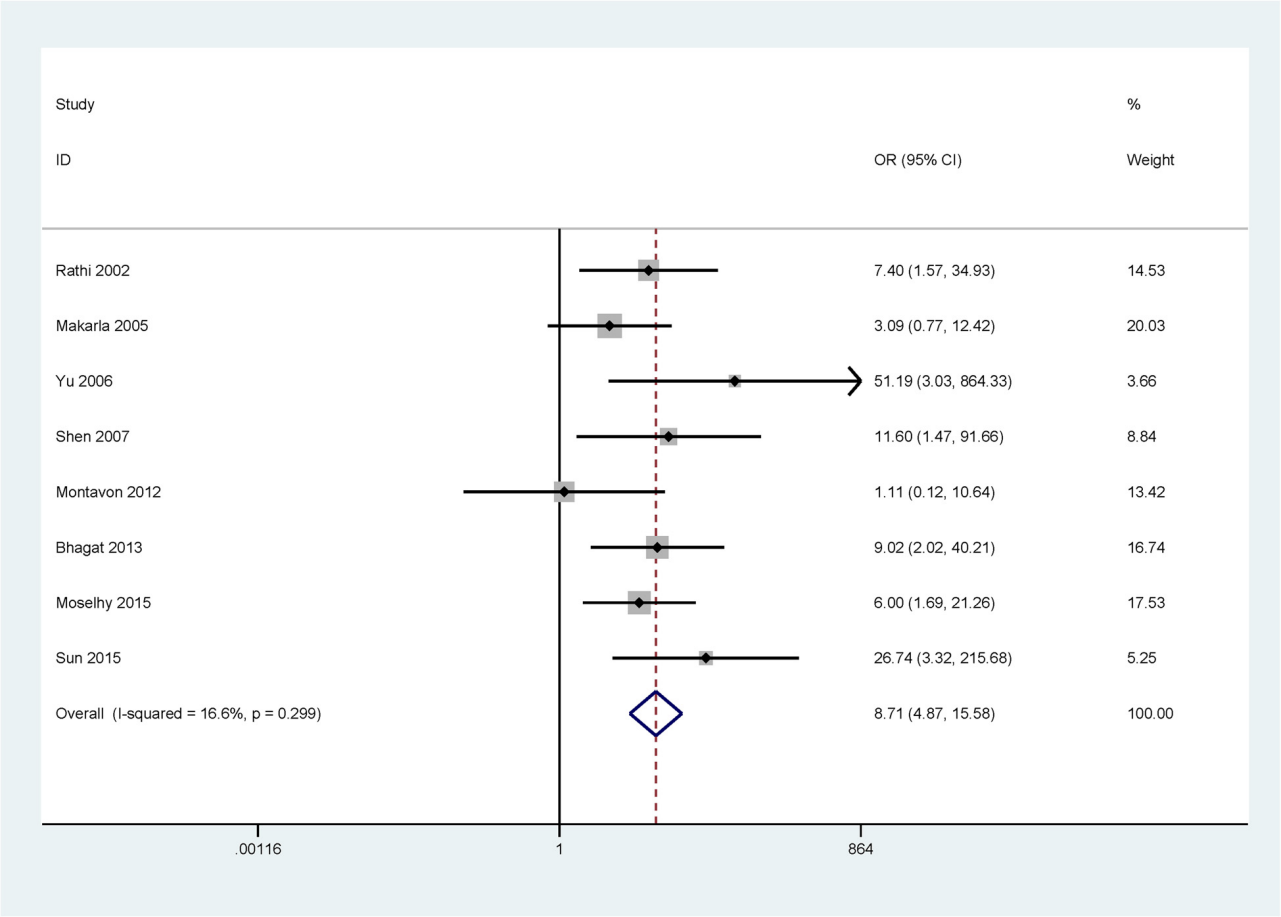
Forest plot of the association between *CDH1* promoter methylation and ovarian cancer

**Fig. 3:**
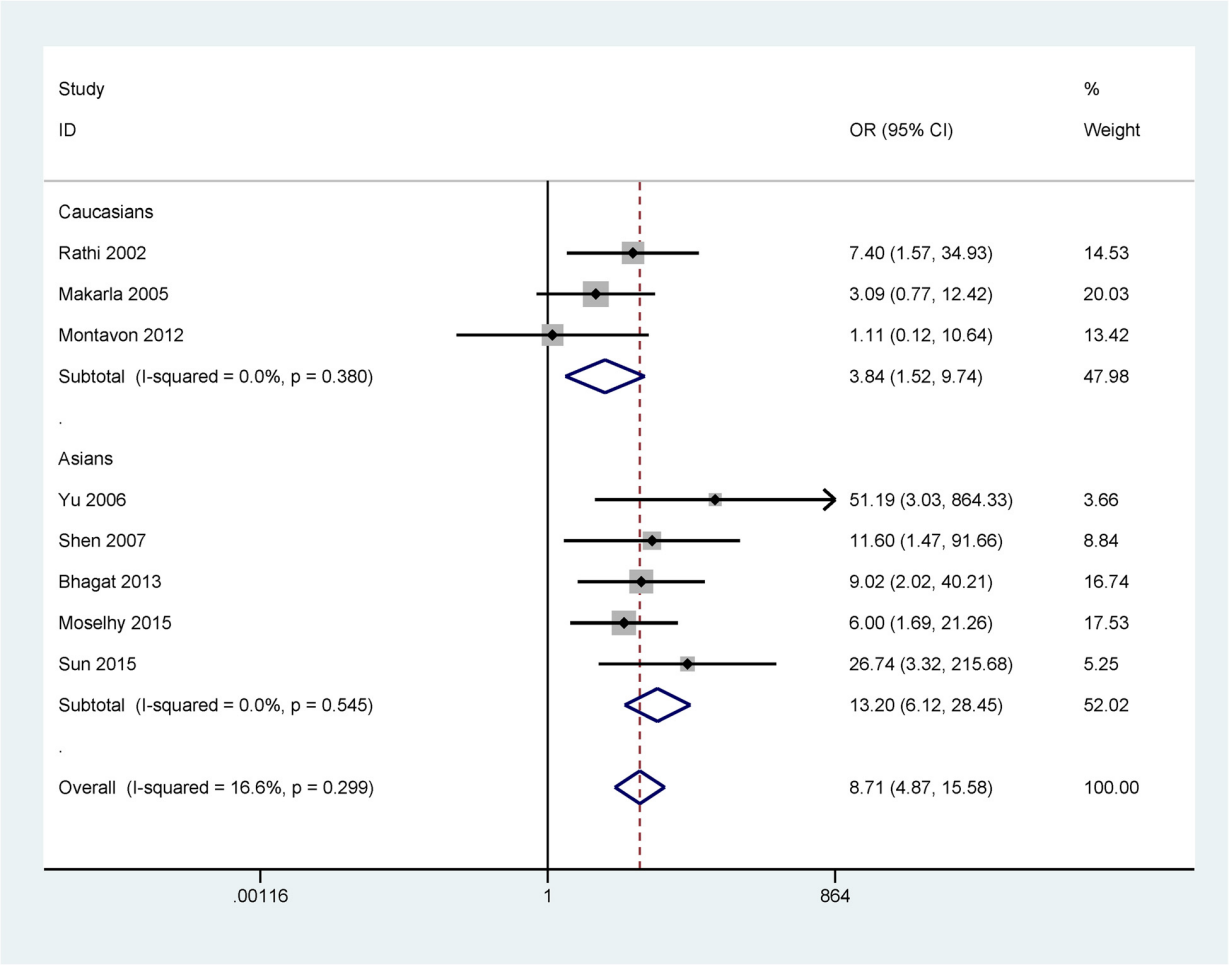
Forest plot of the association between *CDH1* promoter methylation and ovarian cancer based on subgroup analysis of the ethnicity

Significant between-study heterogeneity was not detected (I^2^ = 16.6 % and *P* = 0.299), a fixed-effects model was used. A significant association was observed between *CDH1* promoter methylation and ovarian cancer among 8 studies (OR = 8.71, 95 % CI = 4.87 - 15.58, *P* < 0.001), including 435 malignant tissues from ovarian cancer and 255 nonmalignant tissues (Fig. 2). Subgroup analysis based on the ethnic population showed that the *CDH1* promoter methylation status was significant associated with the risk of ovarian cancer in Asian population and Caucasian population (OR = 13.20, 95 % CI = 6.12 - 28.45, *P* < 0.001; OR = 3.84, 95 % CI = 1.52 - 9.74, *P* = 0.005; respectively) (Fig. 3). No significant association was found in the comparison of ovarian cancer and LMP tumor (OR = 2.40, 95 % CI = 0.86 - 6.76, *P* = 0.096), reporting a total of 109 ovarian cancer patients and 37 low malignant tumor patients in 2 studies (Table 2).

**Table 2:** Summary of the association of *CDH1* promoter methylation and ovarian cancer

	Studies	Overall OR 95CI %	I2; p	*P* value	Cases	Controls	p (Egger’s test)
NMT group	8	8.71(4.87 - 15.58)	16.6 %; 0.299	<0.001	435	255	0.335
Race							
Asians	5	13.20 (6.12 - 28.45)	0.0 %; 0.545	<0.001	285	172	
Caucasians	3	3.84 (1.52 - 9.74)	0.0 %; 0.380	0.005	150	83	
LMT group	2	2.40 (0.86 - 6.76)	0.0 %; 0.512	0.096	109	37	
Clinicopathological features					Patients		
Histology					Stage 1-2	Stage 3-4	
	4	1.41 (0.76 - 2.60)	0.0 %; 0.483	0.273	122	82	0.935
					Patients		
Stage					Serous	Non-serous	
	3	0.55 (0.28 - 1.08)	45.3 %; 0.161	0.082	57	142	0.316

*NMT* nonmalignant tissues, *LMP* low malignant potential tumor

### The association of *CDH1* promoter methylation and clinicopathological features

The associations between *CDH1* promoter methylation and clinicopathological features were further analyzed in the present meta-analysis (Table 2), such as tumor stage (57 early ovarian cancer patients vs. 142 advanced ovarian cancer patients) and tumor histology (122 serous cancer patients vs. 82 non-serous cancer patients), including 3 studies and 4 studies respectively. Between-study heterogeneity was lack (*P* > 0.1), the fixed-effects model was used. The result showed that *CDH1* promoter methylation was not significantly associated with tumor histology and tumor stage (OR = 1.41, 95 % CI = 0.76 - 2.60, *P* = 0.273; OR = 0.55, 95 % CI = 0.28 - 1.08, *P* = 0.082; respectively).

### Publication bias

Egger’s test was performed to estimate the publication bias of included studies. Egger’s test of *CDH1* methylation of cancer versus nonmalignant control showed that there was not any evidence of publication bias (*P* = 0.335). No publication bias was detected in tumor histology and tumor stage (*P* = 0.935 and *P* = 0.316 respectively) (Table 2).

## Discussion

The gene epigenomic regulation of initiation and progression of cancer has two essential components of the molecular mechanism, which are the hypermethylation of tumor suppressor genes and hypomethylation of oncogenes [27–29]. The CpG islands methylation of the promoter is an important reason for loss of gene expression, which can lead to the transcription repression of the gene [30]. Inactivation of *CDH1* by promoter hypermethylation has been observed in several types of cancers, including breast cancer, ovarian cancer and gastric cancer [31–33]. However, the frequency of *CDH1* promoter methylation was inconsistent. Montavon et al. reported that the frequency of *CDH1* promoter methylation was 21.8 % and 20 % in ovarian cancer and nonmalignant ovarian disease respectively [34]. Rathi et al. reported that the frequency of *CDH1* promoter methylation was 28.6 % and 5 % in ovarian cancer tissues and nonmalignant tissues respectively [35]. So the current meta-analysis was performed to identify the association between *CDH1* promoter methylation and ovarian cancer risk.

A total of 9 studies including 485 cancer tissues and 255 nonmalignant tissues were involved in our study. *CDH1* promoter methylation had an increased risk in cancer tissues (OR = 8.71, 95 % CI = 4.87 - 15.58, *P* < 0.001) in comparison with nonmalignant tissues. Subgroup analysis based on the ethnicity suggested that the *CDH1* promoter methylation status was significantly increased risks of ovarian cancer in Asian population and Caucasian population (OR = 13.20, 95 % CI = 6.12 - 28.45; OR = 3.84, 95 % CI = 1.52 - 9.74; respectively). The OR value of Asian population subgroup (OR = 13.20) was higher than that in Caucasian population subgroup (OR = 3.84), suggesting that Asian population can be more susceptible to *CDH1* promoter methylation. However, the results should be interpreted with caution as only small subjects were included in subgroup analyses. No significant association was observed between ovarian cancer and LMP tumor (*P* = 0.096), including a total of 109 ovarian cancer patients and 37 low malignant tumor patients.

We further evaluated the relationships of *CDH1* promoter methylation with clinicopathological features, such as tumor histology and tumor stage. Our findings indicated that the *CDH1* promoter methylation status was not significantly associated with tumor stage and histology. Publication bias was not detected by Egger’s test (all *P* > 0.05).

The current study had some limitations. Firstly, the search strategy was restricted to articles published in English or Chinese. Secondly, the total sample size was not sufficient larger (less than 1000) [36], our results may be lack vigorous power to evaluate the associations between *CDH1* promoter methylation and ovarian cancer risk. Thirdly, based on the limitation of insufficient data, we did not study the *CDH1* promoter methylation status in other clinicopathological features, such as tumor grade, sex status and age etc. Therefore, a meta-analysis including more studies with larger sample size should be necessary to confirm the results in the future.

## Conclusion

*CDH1* promoter methylation is significantly associated with ovarian cancer risk. In addition, the potential association on *CDH1* promoter methylation and some clinicopathological features are still unclear due to the limitation of studies and sample size.

### Ethics approval and consent to participate

Not applicable

### Consent for publication

Not applicable.

### Availability of data and material

All data is available in this paper.
